# Idiopathic Oxalate Nephropathy Leading to End-Stage Kidney Disease: A Case Report

**DOI:** 10.7759/cureus.42402

**Published:** 2023-07-24

**Authors:** Maryam Saleem, Khadija Shahjahan, Hassaan Iftikhar

**Affiliations:** 1 Nephrology, Ohio Valley Nephrology Associates, Owensboro, USA; 2 Nephrology, Washington University School of Medicine, St. Louis, USA; 3 Internal Medicine, Waterbury Hospital, Waterbury, USA; 4 Internal Medicine, Faisalabad Medical University, Faisalabad, PAK; 5 Nephrology, Washington University School of Medicine, Saint Louis, USA; 6 Internal Medicine, Saint Francis Medical Center, Trenton, USA

**Keywords:** end stage kidney disease (eskd), idiopathic hyperoxaluria, primary hyperoxaluria, oxalate nephropathy, aki

## Abstract

Oxalate nephropathy represents a frequently overlooked etiology of renal failure, characterized by the deposition of calcium oxalate crystals within the renal parenchyma. This progressive form of kidney disease is marked by a significant increase in serum creatinine (Cr) level accompanied by evidence of oxalate crystal deposition on renal biopsy causing tubular obstruction and tubular injury leading to fibrosis. In all instances of oxalate nephropathy, examination of stones consistently exhibits multiple birefringent calcium oxalate crystals under polarized light. This case report details the clinical course of a patient who initially presented with progressively worsening renal function and ultimately developed end-stage kidney disease (ESKD) as a consequence of idiopathic hyperoxaluria.

## Introduction

Oxalate nephropathy, though often underrecognized, represents a notable cause of kidney failure, accounting for approximately 1% of native kidney biopsies [1]. Typically presented as either acute kidney injury (AKI) or chronic kidney disease (CKD), the disease carries a grim prognosis, with a high incidence of renal failure needing dialysis within the initial month following diagnosis at a rate as high as 52% [1,2]. Idiopathic hyperoxaluria is a rare entity that is usually associated with recurrent calcium oxalate stones; however, there have been no known cases of end-stage kidney disease (ESKD) from idiopathic hyperoxaluria [3].

## Case presentation

**Figure 1:**
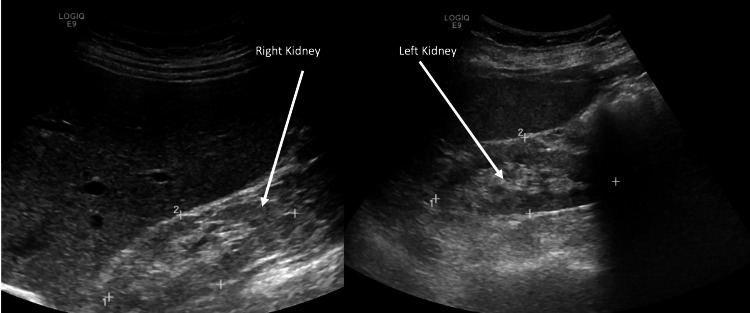
Renal ultrasound with increased echogenicity of renal parenchyma

A 55-year-old female, with a past medical history of asthma, gastroesophageal reflux disease, fibromyalgia, obstructive sleep apnea, and osteoporosis (treated with calcium supplements) presented to the hospital with an abnormal laboratory workup done at the primary care physician's office. The patient had a routine six-month follow-up with the primary care physician a day before the presentation, where the serum creatinine (Cr) was found to be elevated at 5.9 mg/dL (reference range: 0.7-1.3 mg/dL) from a normal baseline of 0.9 mg/dL six months ago. Other than subjective oliguria, the patient was feeling well and had no flank or abdominal pain or urinary symptoms. There was a remote history of sparse ibuprofen use. No nephrotoxic medications were identified in the medication list, and the patient was not on any herbal supplements. An initial workup was obtained for AKI. Urinalysis with microscopy was negative for protein, blood, cells, or casts. Renal ultrasound was unremarkable except for increased cortical echogenicity, as shown in Figure 1. No obstructions or stones were identified.

**Figure 2:**
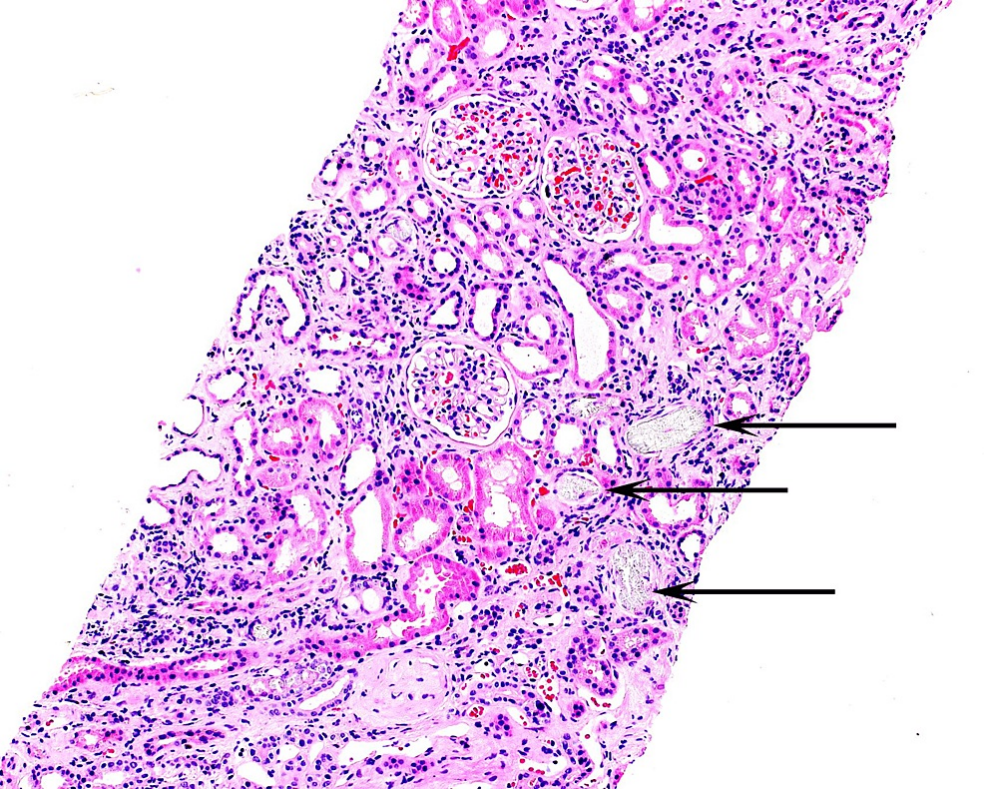
Tubular injury with oxalate crystal deposition as indicated by black arrows Courtesy of Arakana Laboratories

**Figure 3:**
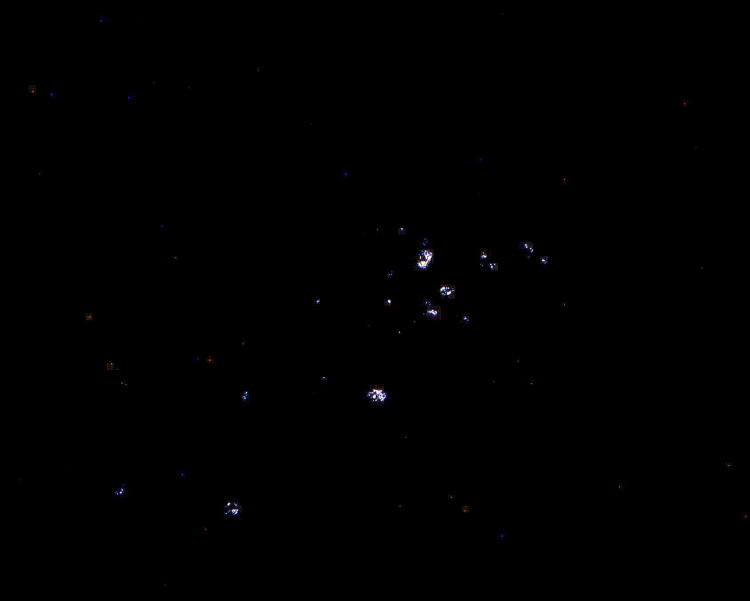
Oxalate crystals with bright birefringence under polarized light Courtesy of Arakana Laboratories

A serological workup was obtained to rule out autoimmune diseases and was negative for anti-nuclear antibody (ANA), anti-double-stranded DNA antibody (dsDNA), and normal serum complement levels. The patient remained non-oliguric, and Cr stayed between 5 mg/dl and 6 mg/dl without any improvement with intravenous fluids. Twenty-four hour creatinine clearance was 8 ml/min. Ultimately, the patient underwent a kidney biopsy for diagnostic clarification as well as prognostication. Kidney biopsy revealed protracted tubular injury (Figure 2) with calcium oxalate deposition in tubules (Figure 3), favoring possible oxalate nephropathy.

The serum oxalate level was 8.9 µmol/L (reference range: <2 µmol/L). Due to a persistently oliguric state, 24-hour urine oxalate testing was deferred. Based on these biopsy findings, the patient was further investigated for higher dietary consumption of oxalate-containing foods such as rhubarb, nuts, spinach, and starfruit, which the patient denied. There was no history of consumption of vitamin C, cyclooxygenase-2 inhibitors, orlistat, ingestion of ethylene glycol, gastric bypass surgery, or malabsorptive disorders. The vitamin B6 level was within normal limits. Primary hyperoxaluria (PH) was considered, and the patient underwent genetic testing, which was also negative for gene mutations, as represented in Table 1.

**Table 1:** Genetic testing for primary hyperoxaluria

Gene Analyzed	Result
AGXT (alanine-glyoxylate aminotransferase)	Negative
GRHPR (glyoxylate reductase–hydroxypyruvate reductase)	Negative
HOGA1 (enzyme 4-hydroxy-2-oxoglutarate aldolase)	Negative

The patient progressed to ESKD a few weeks later and was started on peritoneal dialysis. The cause of hyperoxaluria leading to oxalate nephropathy remains idiopathic.

## Discussion

Renal injury in oxalate nephropathy shares molecular and cellular mechanisms as observed in other crystal-associated diseases [4]. Pathophysiologically, renal inflammation from oxalate crystals usually stems from intraluminal crystal deposition leading to tubular obstruction, especially obstruction of >50% tubules, which can lead to AKI. Moreover, tubular injury can also result from endocytosis and phagosomal destabilization of oxalate crystals with cell necrosis. This causes the release of damage-associated molecular patterns (DAMPs), which activate pattern recognition receptors on other tubular cells, leading to an inflammatory cascade with cytokine release. Finally, calcium oxalate crystals can also get deposited in the interstitium, causing further inflammation and eventually interstitial fibrosis [4-5]. Risk factors include >50 years of age, diabetes mellitus, hypertension, and the use of medications such as angiotensin blockade and diuretics [6]. PH has three types. PH type 1 is the most common type and is caused by a deficiency of the liver-specific enzyme alanine-glyoxylate aminotransferase (AGT), which catalyzes the transamination of glyoxylate to glycine. PH type 2 is caused by a deficiency of glyoxylate reductase-hydroxypyruvate reductase (GRHPR), which catalyzes the reduction of glyoxylate to glycolate and hydroxypyruvate to D-glycerate. In PH type 3, there are defects in the liver-specific mitochondrial enzyme 4-hydroxy-2-oxoglutarate aldolase (HOGA), which is essential in the metabolism of hydroxyproline. Oxalate overproduction is common in these enzyme deficiencies. PH, even though it can present at any age, has a median age of onset of around 5.5 years [7]. Secondary hyperoxaluria either results from enteric hyperoxaluria or the consumption of a high-oxalate diet or oxalate precursors. Causes of enteric hyperoxaluria include bariatric surgery, chronic pancreatitis, jejunoileal bypass, intestinal resection for bowel disease, celiac disease, and Crohn’s disease [8]. In the above-mentioned malabsorption syndromes, calcium (which normally binds oxalate in the bowel) is bound to free fatty acids and is not available for oxalate binding, leading to increased oxalate absorption by the bowel [6]. Several case reports have described the consumption of certain high-oxalate foods such as rhubarb, nuts, black tea, and star fruits, as well as the ingestion of oxalate precursors such as vitamin C and ethylene glycol, leading to oxalate nephropathy [6]. Idiopathic hyperoxaluria has been described in the literature and is usually considered in patients without any known causes of enteric or primary hyperoxaluria. Different mechanisms have been proposed to cause idiopathic hyperoxaluria, such as increased oxalate synthesis and increased absorption, especially on the background of low dietary intake of calcium as well as increased tubular secretion of oxalate [3]. Idiopathic hyperoxaluria is a known risk factor for recurrent kidney stones, and various factors can decrease the risk of stone formation, such as weight loss, decreased dietary consumption of oxalate, and increased urinary volume [9]. There are convincing data that idiopathic hyperoxaluria can lead to nephrolithiasis or nephrocalcinosis, but it has not been known to cause ESKD based on the available data. To the best of our knowledge, this is the first reported case of idiopathic hyperoxaluria leading to ESKD.

## Conclusions

In conclusion, oxalate nephropathy is under-recognized and typically manifests as AKI or CKD. It has a poor prognosis, with a high likelihood of kidney failure within the first few months after diagnosis. Early and accurate diagnosis, along with appropriate management strategies, can potentially reduce intestinal oxalate absorption and the deposition of calcium oxalate crystals in the kidneys. The mechanism behind idiopathic hyperoxaluria leading to ESKD remains unknown, and further research is needed to identify and study such a subset of the patient population.

## References

[1] Buysschaert B, Aydin S, Morelle J, Gillion V, Jadoul M, Demoulin N (2020). Etiologies, clinical features, and outcome of oxalate nephropathy. Kidney Int Rep.

[2] Demoulin N, Aydin S, Gillion V, Morelle J, Jadoul M (2022). Pathophysiology and management of hyperoxaluria and oxalate nephropathy: a review. Am J Kidney Dis.

[3] Glew RH, Sun Y, Horowitz BL (2014). Nephropathy in dietary hyperoxaluria: a potentially preventable acute or chronic kidney disease. World J Nephrol.

[4] Mulay SR, Evan A, Anders HJ (2014). Molecular mechanisms of crystal-related kidney inflammation and injury. Implications for cholesterol embolism, crystalline nephropathies and kidney stone disease. Nephrol Dial Transplant.

[5] Mulay SR, Anders HJ (2017). Crystal nephropathies: mechanisms of crystal-induced kidney injury. Nat Rev Nephrol.

[6] Rosenstock JL, Joab TM, DeVita MV, Yang Y, Sharma PD, Bijol V (2022). Oxalate nephropathy: a review. Clin Kidney J.

[7] Cochat P, Rumsby G (2013). Primary hyperoxaluria. N Engl J Med.

[8] Asplin JR (2016). The management of patients with enteric hyperoxaluria. Urolithiasis.

[9] Schwen ZR, Riley JM, Shilo Y, Averch TD (2013). Dietary management of idiopathic hyperoxaluria and the influence of patient characteristics and compliance. Urology.

